# Microplastics’ Shape and Morphology Analysis in the Presence of Natural Organic Matter Using Flow Imaging Microscopy

**DOI:** 10.3390/molecules28196913

**Published:** 2023-10-03

**Authors:** Soyoun Kim, Yejin Hyeon, Chanhyuk Park

**Affiliations:** Department of Environmental Science and Engineering, Ewha Womans University, Seoul 03760, Republic of Korea

**Keywords:** FlowCam, microplastic, natural organic matter, pre-treatment, wastewater

## Abstract

Ubiquitous microplastics in urban waters have raised substantial public concern due to their high chemical persistence, accumulative effects, and potential adverse effects on human health. Reliable and standardized methods are urgently needed for the identification and quantification of these emerging environmental pollutants in wastewater treatment plants (WWTPs). In this study, we introduce an innovative rapid approach that employs flow imaging microscopy (FlowCam) to simultaneously identify and quantify microplastics by capturing high-resolution digital images. Real-time image acquisition is followed by semi-automated classification using customized libraries for distinct polyethylene (PE) and polystyrene (PS) microplastics. Subsequently, these images are subjected to further analysis to extract precise morphological details of microplastics, providing insights into their behavior during transport and retention within WWTPs. Of particular significance, a systematic investigation was conducted to explore how the presence of natural organic matter (NOM) in WWTPs affects the accuracy of the FlowCam’s measurement outputs for microplastics. It was observed that varying concentrations of NOM induced a more curled shape in microplastics, indicating the necessity of employing pre-treatment procedures to ensure accurate microplastic identification when utilizing the FlowCam. These observations offer valuable new perspectives and potential solutions for designing appropriate treatment technologies for removing microplastics within WWTPs.

## 1. Introduction

The growing concern over environmental pollution caused by microplastics has prompted significant attention from both societies and governments [1,2,3]. Microplastics are polymeric particles ranging from 1 µm to less than 5 mm in any dimension [4,5], and are classified as primary and secondary microplastics according to the production process [6]. The former are intentionally manufactured into micro-sizes, such as granules in cosmetics and personal care products, and the latter are caused by the fragmentation of relatively large plastics induced by environmental damage such as photodegradation, weathering processes, or pyrolysis [5,7]. Microplastics in urban water sources exhibit considerable variations in concentration, polymer type, and size [8,9,10,11]. Notably, the wastewater entering the treatment facility contains various types of microplastics, such as polyethylene (PE), polyamide (PA), polypropylene (PP), and polystyrene (PS) [12]. These microplastics are partially removed through the common wastewater treatment process including a pretreatment, and a primary and secondary treatment, showing a removal efficiency of over 88%; but a large amount of microplastics is still discharged into the ocean [13,14,15]. Therefore, an additional tertiary treatment for particle separation is required to completely remove microplastics from wastewater effluent. Recent studies have consistently shown that membrane-based separations can achieve the most efficient and stable removal performance, ranging from 90 to 99.9%. This is achieved by separating particles through size exclusion using dense pores, in contrast to other tertiary treatment processes such as disc filtration (DF, 80% to 98%), rapid sand filtration (RSF, 50% to 98%), ozone plant (85% to 96%), and dissolved air flotation (DAF, 60% to 85%) [16,17]. In addition, existing studies have shown that membrane bioreactor (MBR) processes treat 99.9% of microplastics in the influent wastewater [18,19,20]. Extensive in situ investigations have revealed that the microplastics found in current wastewater treatment plants (WWTPs) are predominantly aged, with roughened surfaces and varied types of oxygen-containing functional groups (i.e., carbonyl and hydroxyl) [21]. Due to their high specific surface areas and small particle sizes, microplastics are recognized as crucial agents facilitating the transport of both inorganic and organic pollutants in urban water systems, including heavy metals, antibiotics, and persistent organic pollutants [22,23,24,25]. Consequently, a sophisticated sampling and pre-treatment procedure has been introduced to facilitate the analysis of microplastics within WWTPs [26,27,28].

At present, substantial scientific efforts are being directed toward the development of a rapid and precise alternative to the labor-intensive manual microscopy methods [29,30,31,32]. Various analytical techniques are currently being employed for the chemical identification of microplastics, including micro-Fourier transform infrared (μ-FTIR) spectroscopy and Raman micro-spectroscopy (μ-Raman) [33,34,35]. μ-FTIR is a versatile technique, enabling chemical imaging over large areas of membrane filters using focal plane array (FPA) detectors [36,37]. This FTIR imaging form allows for rapid measurements with high resolution, limited to approximately 10 μm by diffraction in μ-Raman [38,39,40]. To effectively utilize this technique, the removal of organic contaminants that adhered to the surface of microplastics is often required. This can be achieved through a variety of available treatments [41,42]. These microscopic assessments of microplastics conventionally rely on straightforward length and width measurements [43]. In contrast to other spectroscopic methods, flow imaging microscopy (FlowCam) offers the advantage of fully automated microplastic analysis [44,45]. Additionally, this method can identify small and inconspicuous microplastics that might be overlooked during manual analysis [46]. Through image analysis, it becomes possible to provide high-resolution morphological details of microplastics, thereby enhancing product quality and performance as well as obtaining accurate measurements of size and quantity.

This study proposes a FlowCam approach that not only captures a permanent digital record of samples but also rapidly acquires multiple measurements of microplastics, reducing the time and labor required for microplastic identification. The technique automatically captures high-resolution digital images of microplastics from samples of flowing water streams to quantify the microplastics and extract the relevant parameters from the individually captured particle images in real time, resulting in the visualization of microplastics. In addition, this machine has a wide size range of 0.9–1000 μm and can minimize the loss of particles by injecting water samples directly into the instrument. Due to these advantages, this technique has found widespread use in microbiology for observing microorganisms, especially phytoplanktons. Their number and growth in various bodies of water have been effectively identified by systematically analyzing the unique appearance of each species [47,48,49]. Moreover, with this technique, there is no need for chemical degradation during sample preparation, which means no damage occurs to microplastics. This makes it particularly suitable for characterizing microplastics. The FlowCam, equipped with VisualSpreadsheet^®^ software version 5.9.1.78, is employed for its capability to semi-automatically classify collected images using pre-defined image libraries and statistical pattern-recognition algorithms [50]. Our custom-designed image libraries and algorithms rely on morphological analysis to discern pertinent shape-related information. Given the substantial similarity between these requirements and those for microplastic analysis, we have chosen to adapt this image library for the identification of microplastics within the collected image datasets. Furthermore, a comprehensive investigative framework has been established to provide full information on the characterized microplastics. This includes outputs such as length, area, volume, shape-based, and fiber-based parameters, as well as the total count of microplastics.

With the aim of automatically evaluating the fate, transport, and retention of microplastics within WWTPs, we introduced the FlowCam integrated with its accompanying software. This setup enables the identification of typical microplastics through high-resolution images acquired in real-time. Specifically, we conducted a comparative analysis involving two different types of microplastics (PE and PS). We automatically cropped individual particle images and evaluated the effectiveness of automated classifications by manually checking the FlowCam’s measurement outputs. Furthermore, we explored the necessity of microplastic pre-treatment and extraction to ensure their accurate identification using the FlowCam. Our investigation aimed to ascertain the potential prerequisites for achieving precise microplastic identification. Additionally, a systematic examination was undertaken by employing the FlowCam to gain insights into how the presence of natural organic matters (NOMs), which are ubiquitous in aquatic environments, influences the reliability of the FlowCam’s measurement outputs for microplastics.

## 2. Results and Discussion

### 2.1. Identification of Microplastics by the FlowCam

VisualSpreadsheet^®^, a powerful multifactor data analysis software program, can remarkably accelerate the scientific research process by sorting, filtering, quantifying, and classifying actual images. Furthermore, it has the capacity to track how response variables are influenced, whether individually or jointly, by various factors. In this study, to ensure the exclusion of artifacts such as dirt on the flow cell wall, bubbles, contaminants, or backgrounds with different shapes and colors compared to the target microplastics, a series of steps were undertaken before the initial measurements of each microplastic. The flow rate was adjusted to 5 mL/min, and the imaging rate was increased to 20 frames per second. This setup allowed the device to simultaneously capture a larger number of microplastics with greater accuracy during the measurement process. Additionally, real-time monitoring of the analyzing process was possible by observing the camera view of the flow cell, thereby ensuring thorough microplastic analysis. If any inaccuracies were detected during analysis, we would repeat the measurement, typically conducting a set of three measurements, which were later averaged to ensure data reliability. 

**Figure 1:**
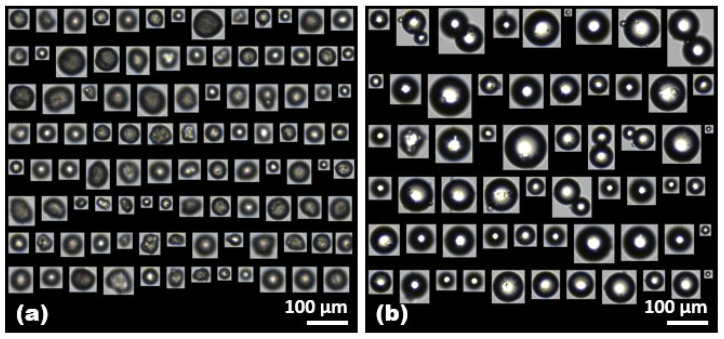
Representative images of (**a**) PE and (**b**) PS microplastics captured through FlowCam analysis by semi-automatic image collection with the VisualSpreadsheet^®^ software. The images in (**a**,**b**) represent 13% and 25% of the total number of selected microplastics in the sample (1 mL), respectively.

Here, the counted images of PE and PS microplastics were primarily regarded as a suitable response variable, and several meaningful variables were identified with semi-automated classification by selecting representative factors such as morphological parameters. Figure 1 shows the representative images of the PE and PS microplastics captured through FlowCam analysis by semi-automatic image collection with the VisualSpreadsheet^®^ software. These images correspond to 13% and 25% of the total captured PE and PS microplastics, achieved by applying advanced custom acquisition filters following the establishment of the reference library for individual microplastics. The auto-classification filters were applied to quantify the captured PE and PS microplastics as they passed through a sample volume of 50 mL at a concentration of 50 mg/L for each microplastic (Table 1). Notably, at the same concentration, the FlowCam identified a greater number of PE microplastics per sample, potentially due to the larger average size of PS microplastics. Distinct morphological differences between the captured images of PE and PS microplastics revealed that the PE microplastics were irregularly shaped, whereas the PS microplastics were relatively spherical, often appearing as pairs adhering to each other. Therefore, a comprehensive analysis of the classified unique properties associated with each microplastic was systematically conducted, providing a clear insight into the transport of microplastics during wastewater treatment processes.

**Figure 2:**
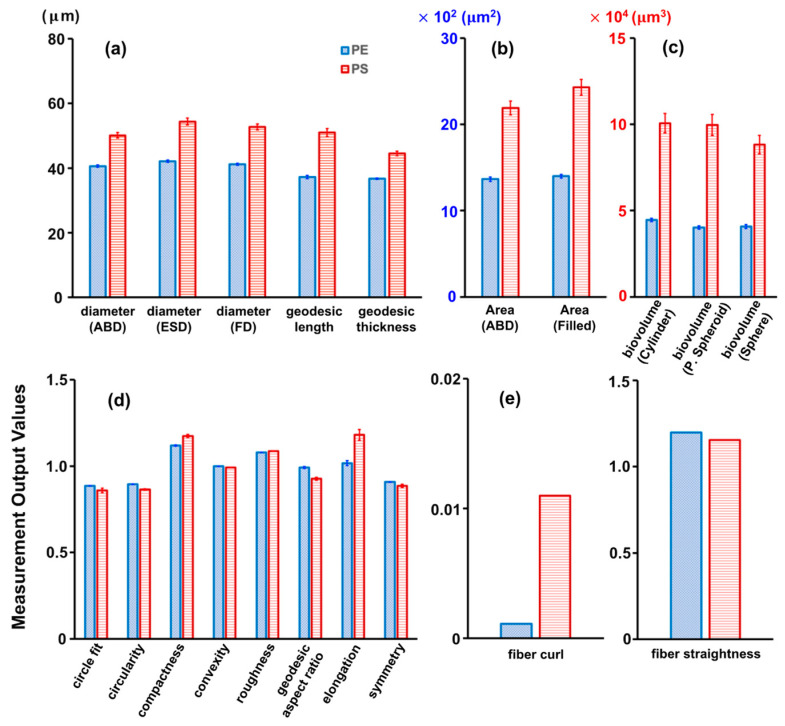
Comparison of the PE and PS microplastic properties in (**a**) 1D, (**b**) 2D, (**c**) 3D, (**d**) shape-based, and (**e**) fiber-based measurement parameters.

In addition to capturing images of microplastics, the instrument’s software automatically collected over 40 particle properties per image, many of which provided valuable insights into the structure and behavior of microplastics in natural and engineered systems. Notably, variations in one- (1D), two- (2D), and three-dimensional (3D), shape-based, and fiber-based measurement outputs were evident when comparing PE and PS microplastics against the samples (Figure 2). The most commonly used measures for indicating microplastic size includ area-based diameter (ABD), equivalent spherical diameter (ESD), filled diameter (FD), geodesic length, and thickness. These values were obtained by modeling the particle as a rectangle and computing its length and thickness (Figure 2a). Additional properties were available for images acquired within the software, such as the pixel count converted to area (Area ABD) and the area encompassed by the particle edge and all pixels inside the edge (Area (Filled)) (Figure 2b). Biovolume, which estimates the volume of microplastics in a unit of the sample, was determined based on the geometric shape that most closely resembled the microplastic image. This volume estimation can be based on a cylinder, prolate spheroid (P-spheroid), or spherical shape (Figure 2c). These 1D, 2D, and 3D measurement outputs tended to be larger for the PS microplastics than for the PE microplastics. The study also introduced additional measurement outputs, including a set of shape-based measures derived from simple calculations using the 1D or 2D measurement outputs. Remarkably, there were no significant differences in estimated shape-based outputs between the two types of microplastics, except for their geodesic aspect ratio and elongation (Figure 2d). Elongation, which is the inverse of the geodesic aspect ratio, values close to one for the PE microplastics indicated a circular shape, whereas similar values for the PS microplastics indicated more elongated particles. Similarly, the two types of microplastics demonstrated significant differences in the fiber-based measurement outputs. Additionally, using both Feret- and geodesic-based measurements led to additional useful descriptors, such as fiber curl and fiber straightness (Figure 2e). The fiber curl value for the PS microplastics showed a value over 10 times greater than that for the PE microplastics, demonstrating that the former possessed a more pronounced curled shape.

### 2.2. Effect of Humic Acid (HA) on the Analysis of PE Microplastics

**Figure 3:**
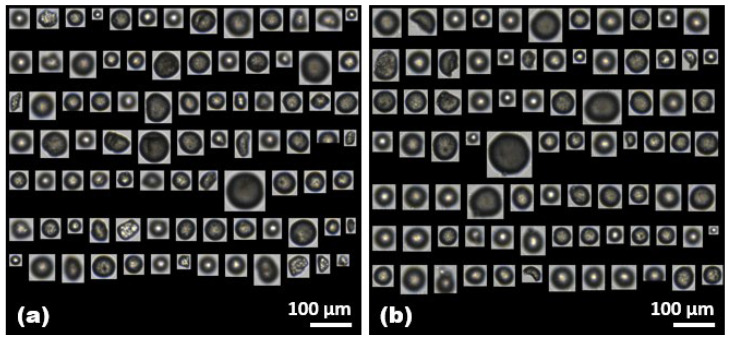
Representative captured images of (**a**) the PE microplastics with an HA concentration of 50 mg/L and (**b**) the PE microplastics with an HA concentration of 500 mg/L. The images in (**a**,**b**) represent 12% and 13% of the total number of selected microplastics in the sample (1 mL), respectively.

In wastewater treatment processes, microplastics coexist with various organic compounds. Most microscopic and spectroscopic methodologies for quantifying microplastics involve relatively long analysis times due to chemical digestion. These methods are highly sensitive to interference from the organic environmental matrix adsorbed onto microplastics. Employing the FlowCam for microplastic analysis can enable rapid and efficient image analysis, potentially simplifying pre-treatment procedures. Therefore, the impact of HA, a representative organic compound present in the water environment, on the FlowCam’s analysis of microplastics was systematically investigated. The PE microplastics at a concentration of 50 mg/L were combined with the HA at concentrations of 50 mg/L and 500 mg/L, respectively, and the effects of the HA on the FlowCam’s analysis of microplastics were subsequently assessed. The visual examination of the representative captured images showed no significant differences as the HA concentrations increased (Figure 3).

Considering that HA adsorption onto microplastics might alter the 1D measurement outputs, particularly those based on geodesic and Feret measurements, a detailed investigation was conducted to determine the influence of HA concentrations (Table 2). These values were also used to investigate the application of the area or volume output to represent microplastic images. The geodesic length outputs were derived by modeling the particle as a rectangle that matched both the area (filled) and perimeter measures, which were both directly computed from the pixels of the image. The Feret measurements corresponded to the perpendicular distance between two parallel tangents touching opposite sides of the particle. As expected, both geodesic and Feret lengths slightly increased with HA concentration, as did the perimeter calculated from the length of the particle edge and the convex perimeter. Consequently, it can be inferred that the presence of HA leads to a slight overestimation of a microplastic’s length and perimeter compared to the absence of HA. Therefore, pre-treatment involving the removal of organic compounds is essential for the accurate identification of microplastics using the FlowCam.

**Figure 4:**
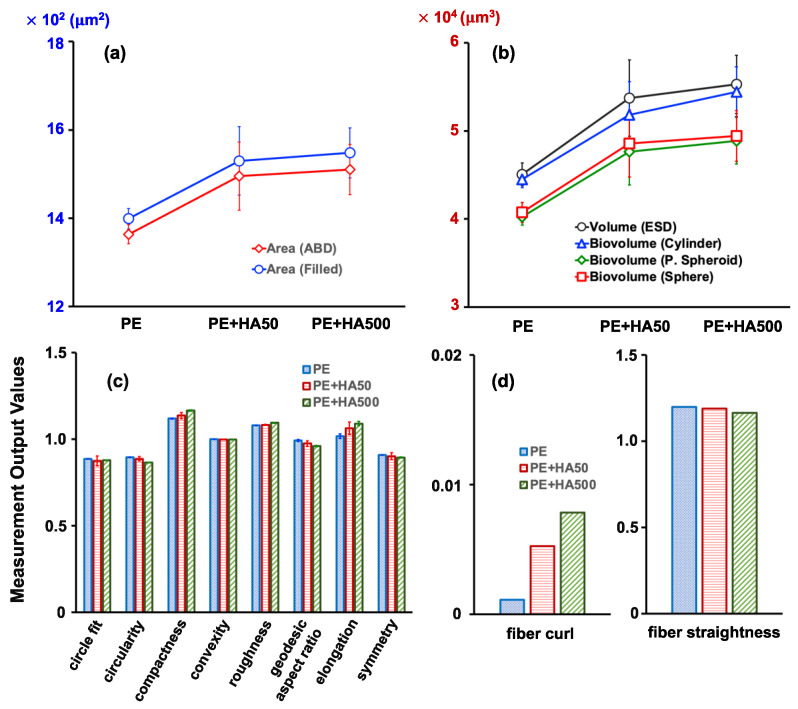
Effect of HA concentration on the PE microplastic’s properties for (**a**) 2D, (**b**) 3D, (**c**) shape-based, and (**d**) fiber-based measurement parameters.

Additional measurement outputs including 2D and 3D parameters were further investigated to elucidate the impact of HA adsorption on image analysis (Figure 4). Both the Area ABD and the Area (Filled) showed a slight increase in the presence of HA, suggesting that pixels are influenced by HA (Figure 4a,b). This trend was also observed for biovolume measures, indicating that HA needs to be removed before the FlowCam’s microplastic analysis. Several morphological factors did not experience significant changes due to the presence of HA (Figure 4c). However, the fiber curl descriptor appeared to be particularly affected, indicating a substantial impact of HA. As the concentration of HA increased, more strongly curled microplastics tended to be created (Figure 4d). Fiber straightness refers to the ratio between Feret length and geodesic length. The increase in fiber curl with increasing HA concentrations was accompanied by a decrease in fiber straightness. This can be attributed to the fact that the rate of change in geodesic length was greater than that of Feret length, as indicated in Table 2. It was also noted that HA adsorbed onto the surface of the PE microplastics, which could potentially alter the shape of the microplastics without affecting their physical structure.

### 2.3. Effect of Bovine Serum Albumin (BSA) on the Analysis of PE Microplastics

**Figure 5:**
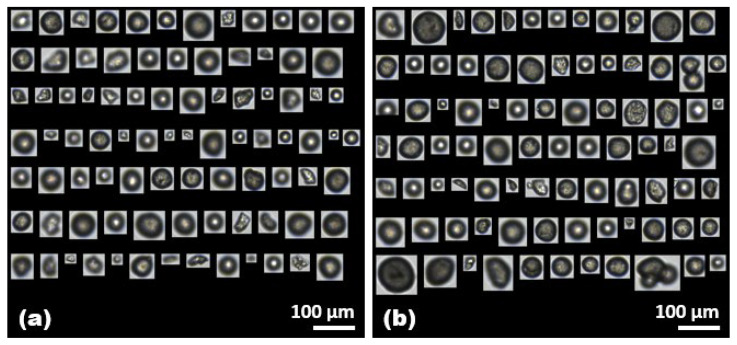
Representative captured images of (**a**) the PE microplastics with a BSA concentration of 50 mg/L and (**b**) the PE microplastics with a BSA concentration of 500 mg/L. The images in (**a**,**b**) represent 12% and 13% of the total number of selected microplastics in the sample (1 mL), respectively.

BSA has been selected as a model protein in scientific investigations designed to clarify the reaction between microplastics and NOMs; it is mainly composed of humic substances, proteins, and carbohydrates/polysaccharides. Figure 5 shows the representative images of the PE microplastics captured by the FlowCam in the presence of BSA at concentrations of 50 mg/L and 500 mg/L, respectively. When comparing two captured images, it could be inferred that there was no significant difference in the changes on the surface surrounding the microplastics. In addition to capturing images of microplastics, the image outputs were semi-automatically sorted in VisualSpreadsheet^®^ to understand how the presence of BSA influenced the adsorption of various forms of microplastics.

Interestingly, when the microplastics coexisted with the BSA at a concentration of 50 mg/L, there were no significant changes in the geodesic length, Feret length, perimeter, or convex perimeter (Table 3). However, when the BSA concentration increased to 500 mg/L, all these values exhibited substantial increases, along with an increase in the standard deviation. This indicates that, at higher BSA concentrations, the protein adsorption onto the microplastic’s surface leads to an elevation in the 1D measurement outputs observed through the FlowCam. Consequently, it can be inferred that solutions with low protein concentrations might allow for FlowCam analysis without an additional pre-treatment procedure. However, wastewater containing high protein concentrations would likely require pre-treatment to remove the protein before conducting a FlowCam analysis for accurate microplastic characterization. The BSA coating on the microplastic’s surface would be unstable and easily fall off during the adsorption due to the weak binding force induced by the larger molecular size and low carboxyl content [51].

**Figure 6:**
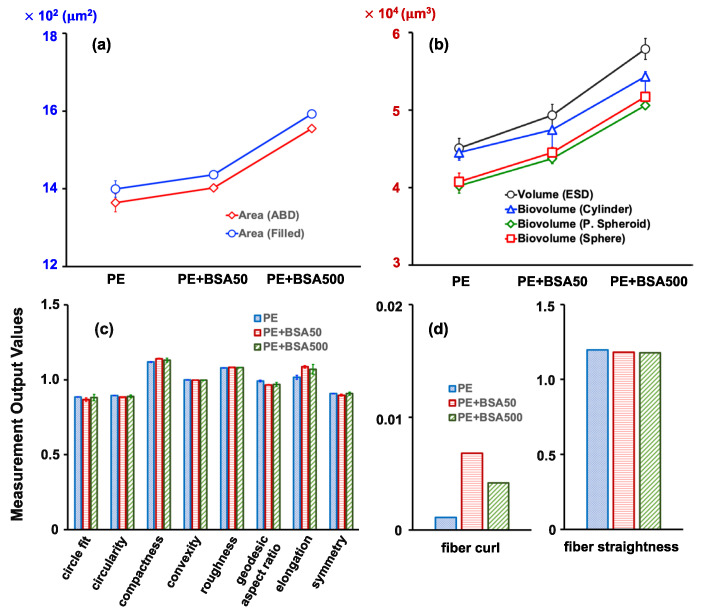
Effect of the BSA’s concentration on the PE microplastic’s properties for (**a**) 2D, (**b**) 3D, (**c**) shape-based, and (**d**) fiber-based measurement parameters.

A comparison of the values representing 2D and 3D measurement outputs revealed distinct outcomes from those representing 1D measurement outputs (Figure 6). Even in the presence of the low BSA concentration of 50 mg/L, both the Area ABD’s and the Area (Filled)’s values increased, and this increase was even more pronounced at the BSA concentration of 500 mg/L (Figure 6a). The biovolume values representing the 3D measurement outputs increased considerably with the BSA concentration, regardless of the assumed shape (Figure 6b). The BSA interfered with the measurement of the microplastics’ area and volume, highlighting the need for a pre-treatment procedure. The BSA, being a large protein with a complex three-dimensional structure, can physically adsorb onto the surface of microplastics through various non-covalent interactions, including van der Waals forces, hydrophobic interactions, and electrostatic attractions. Notably, microplastics may feature diverse functional groups on their surfaces, such as hydroxyl and carboxyl groups, while the BSA contains amino acid residues with varying functional groups that can form hydrogen bonds or other chemical interactions with these surface groups. This interaction can lead to alterations in the surface properties and shape of the microplastics. While other shape-based measurement parameters exhibited minimal differences, the fiber-based measurement parameters, similarly to the effect observed with the HA, showed a substantial increase in fiber curl values in the presence of BSA (Figure 6c,d). The concurrent increase in compactness values, exceeding 1.0, further supported the evidence for the observed increase in fiber curl values when the BSA was present. Proteins like BSA are often hydrated in aqueous solutions. Consequently, when they adhere to the surface of microplastics, they may carry water molecules with them. This hydration layer can influence the physical properties of the microplastics, potentially leading to swelling or shape changes. This suggests that the BSA contributes to microplastics adopting a more curled shape, underscoring the importance of BSA removal through a pre-treatment to ensure the accurate analysis of microplastics’ shapes using the FlowCam.

## 3. Materials and Methods

### 3.1. Microplastics and NOMs

Spherical PE microplastics, with diameters ranging from 10 to 106 μm (Cospheric LLC, Santa Barbara, CA, USA), were initially soaked in 95% (*w*/*w*) ethanol (Samchun Pure Chemical Co., Ltd., Pyeongtaek, Republic of Korea) for 24 h at a room temperature of 20.8 ± 0.6 °C. They were then subjected to three rinses with deionized (DI) water (Direct-Q^®^ 3 Water Purification System, Millipore Corp., Billerica, MA, USA) to remove contaminants. Next, the PE microplastics were dried in an oven at 40 °C for 24 h and finally stored in a glass bottle. The residual ethanol would have completely evaporated during these procedures owing to its high volatility, thus ensuring that it had no impact on the microscopic analysis of the microplastics. PS microplastics (Sigma–Aldrich, St. Louis, MO, USA) with particle sizes corresponding to the 200–400 mesh, which aligns with the 0.035–0.074 mm range of US standard sieves (ASTM E11), were also utilized. The particle size distribution of each type of microplastic was assessed using a particle size distribution analyzer (Mastersizer 3000, Malvern Panalytical Ltd., Malvern, UK) to confirm the validity of the fractions provided by the manufacturer.

HA and BSA have frequently served as representative humic substances and proteins in research investigating the adsorption of NOMs to surface interfaces. Both substances are significant hydrophilic byproducts resulting from the biological treatment and advanced oxidation processes in WWTPs. Previous studies have demonstrated that microplastics exhibited a strong affinity for organic substances, including HA and BSA, binding through oxygen-containing functional groups such as carboxyl, phenolic hydroxyl, and aliphatic groups [32,46]. Test samples were prepared by introducing HA (technical grade, 30 kDa, Sigma–Aldrich, Buchs, Switzerland) or BSA (67 kDa, Sigma–Aldrich, St. Louis, MO, USA) into each microplastic suspension at concentrations of 50 and 500 mg/L. Due to its higher carboxyl content and lower molecular weight, HA offers a greater number of binding sites, making it capable of forming a more stable compound with microplastics compared to BSA. This analysis was conducted to investigate the impact of NOMs on the identification of microplastics using the FlowCam. To determine the actual concentration of organic matter within the samples, the total organic carbon (TOC) was analyzed using a TOC analyzer equipped with an auto sampler ASI-L (TOC-LCPH, Shimadzu Corp., Kyoto, Japan).

### 3.2. FlowCam Apparatus

**Figure 7:**
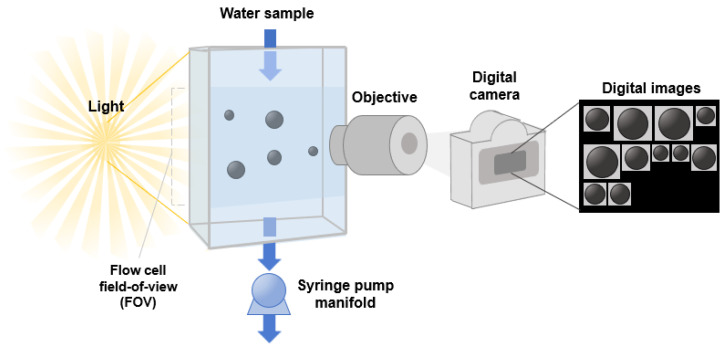
Schematic diagram of flow imaging microscopy (FlowCam) apparatus equipped with flow cell, microscope, and high-speed digital camera.

The FlowCam (FlowCam 8100, Yokogawa Fluid Imaging Technologies, Inc., Scarborough, ME, USA) is an automated imaging analysis system that consists of a combined microscope, digital camera, and flow cytometer to digitally image, count, and measure microplastics (Figure 7). The use of field-of-view (FOV) flow cells, capable of measuring particles within the range from 2 μm to 1 mm, allows for the complete width of the flow cell to be captured by a digital camera. The FlowCam system provides four objective lenses (2×, 4×, 10×, and 20×), each compatible with various flow cell sizes and types. The specific combination of objective lens and flow cell has a direct impact on the quality of the captured images. Given the size characteristics of the PE and PS microplastics under investigation, we opted for a 10× magnification objective lens paired with an FOV100 flow cell from the available options. This particular configuration is optimized for the analysis of microplastics within the 1–100 μm range, making it well-suited for obtaining high-resolution digital images of the PE and PS microplastics in this study. This setup significantly enhances the comprehensive detection and capture of microplastics present in the sample. Controlled through the associated VisualSpreadsheet^®^ software version 5.9.1.78, the FlowCam manages the sample processing, the image capture and cropping, and the extraction of microplastic parameters from each cropped image. The software also functions as a user interface for image viewing and sorting. The FlowCam, in combination with this software, facilitates the automatic classification of collected images by employing custom-defined image libraries.

Prior to each measurement, we ensured the cleanliness of the fluid path and flow cell by rinsing them with DI water. Following this, we introduced 50 mL of the prepared sample into the inlet of the device. The sample was then pumped through a flow cell positioned in front of a microscope lens, which was connected to a digital camera. This entire process was conducted at a controlled flow rate of 5 mL/min for 10 min, encompassing both backgrounds’ calibration. During this calibration, the background intensity was adjusted to a value of 175, as recommended by the manufacturer, to effectively distinguish between the particles and background elements. The particle images within the water sample were captured in the auto-image mode, which involves imaging at timed intervals, with an image rate of 20 frames per second. For the sample analysis, a total of 50 mL of solution was loaded into the sample loader. Initially, 20 mL was run to prime the system, followed by data acquisition for the subsequent three batches of 10 mL. To ensure accuracy, each sample was subjected to three individual measurements, and this process was repeated three times (*n* = nine). The accompanying VisualSpreadsheet^®^ software version 5.9.1.78, supplied with the instrument, was used for data analysis. A filter was implemented to exclude artifacts such as dirt on the flow cell wall, contaminants, and bubbles in order to minimize the capture of non-target microplastics during the software’s auto-classification process. The software itself automatically extracted up to 40 unique morphological properties from each image, several of which hold potential value for understanding the fate and transport of microplastics within WWTPs [44].

## 4. Conclusions

We have identified a key set of characteristics using the FlowCam to image microplastics with the applied principles in order to produce informative methods. Using the FlowCam with the VisualSpreadsheet^®^ software, which collects over 40 particle properties per image, and performing a systematic investigation allowed us to achieve the rapid analysis of the microplastics present in various wastewater samples. When comparing the PE and PS microplastics commonly detected in wastewater, it was observed that, at the same concentration, the relatively smaller-sized PE microplastics were more frequently measured. A comparison of 1D, 2D, and 3D measurement outputs revealed substantial differences in length, volume, and shape. An investigation of the influence of the organic substances present in wastewater, such as HA and BSA, on the FlowCam’s analysis of microplastics showed that the measurement outputs of the microplastic’s length and volume increased significantly with the concentrations of HA and BSA. Compared to the HA, the BSA had a minor effect on the FlowCam’s measurement outputs at the low concentration because it was unstable due to the weak binding force formed by its smaller number of carboxyl groups and larger molecular weight. In particular, it was observed that both the HA and the BSA induced microplastics with a more curled shape, suggesting that HA and BSA may achieve different aggregation/agglomerate states by using their functional groups to bind with the microplastics. To accurately analyze the morphological characteristics of microplastics, it appears reasonable to conduct an analysis through the FlowCam after a suitable pre-treatment procedure to remove HA and BSA. Future studies should continue these comparisons of measurement outputs, but statistical tests should be used systematically to compare the relative differences in each FlowCam measurement output.

## Figures and Tables

**Table 1:** Count-based measurements of PE and PS microplastics identified by the FlowCam in combination with the VisualSpreadsheet^®^ software. A total sample volume of 50 mL with PE and PS microplastic concentrations of 50 mg/L was introduced into a flow cell within the FlowCam instrument.

	Trial 1 (Number/mL)	Trial 2 (Number/mL)	Trial 3 (Number/mL)	Average (Number/mL)
PE	798 ± 70	882 ± 22	843 ± 70	841 ± 42
PS	236 ± 17	334 ± 18	285 ± 9	285 ± 49

**Table 2:** Geodesic- and Feret-based length and perimeter measurement outputs obtained by modeling the PE microplastic as a rectangle in the absence and presence of HA. The PE microplastic concentration is 50 mg/L, and the HA concentrations are 50 mg/L or 500 mg/L.

	Length (Geodesic) (μm)	Length (Feret) (μm)	Perimeter (μm)	Convex Perimeter (μm)
PE	37.24 ± 0.50	44.56 ± 0.35	147.88 ± 1.25	137.06 ± 1.05
PE with HA50	39.12 ± 1.00	46.32 ± 0.96	153.44 ± 3.35	141.93 ± 3.21
PE with HA500	40.25 ± 0.73	46.55 ± 0.88	156.24 ± 2.65	142.92 ± 2.54

**Table 3:** Geodesic- and Feret-based length and perimeter measurements obtained by modeling the PE microplastic as a rectangle in the absence and presence of BSA. The PE microplastic concentration is 50 mg/L, and the BSA concentrations are 50 mg/L or 500 mg/L.

	Length (Geodesic) (μm)	Length (Feret) (μm)	Perimeter (μm)	Convex Perimeter (μm)
PE	37.24 ± 0.50	44.56 ± 0.35	147.88 ± 1.25	137.06 ± 1.05
PE with BSA50	37.99 ± 0.29	44.74 ± 0.15	148.32 ± 0.12	137.09 ± 0.05
PE with BSA500	40.12 ± 0.75	46.98 ± 0.63	156.05 ± 1.15	144.40 ± 0.75

## Data Availability

Data will be made available on request.
